# *In situ* neuronal regeneration: the potential of astrocytes

**DOI:** 10.1093/lifemedi/lnad036

**Published:** 2023-10-03

**Authors:** Rongbang Tan, Reiner F Haseloff, Yuqian Mo, Jingjing Zhang

**Affiliations:** Affiliated Hospital of Guangdong Medical University & Zhanjiang Key Laboratory of Zebrafish Model for Development and Disease, Guangdong Medical University, Zhanjiang 524001, China; Leibniz-Forschungsinstitut für Molekulare Pharmakologie, Berlin 13125, Germany; School of Public Health, Guangdong Medical University, Dongguan 523808, China; Affiliated Hospital of Guangdong Medical University & Zhanjiang Key Laboratory of Zebrafish Model for Development and Disease, Guangdong Medical University, Zhanjiang 524001, China

Astrocytes are predominant glial cells in the central nervous system (CNS) that play key roles in maintaining its homeostasis. Their functions vary from nourishing neurons to regulating extracellular ion homeostasis, cerebral blood flow (CBF), forming glial scarring, and repairing damage. Though astrocytes help maintaining the blood–brain barrier (BBB) with endothelial cells, they are not required in early BBB formation since the BBB functions before astrocytes arise. Astrocytes are generated during the first postnatal week and their number increases six to eight times within the following 2 weeks in the rodent cortex. By postnatal day 20, astrocyte end-feet cover the cerebrovascular walls almost completely, thus regulating CBF and nutrition transport from peripheral blood to CNS to meet neuronal excitation requirements.

Astrocytes are the most abundant and functionally complex glial cells in the CNS. Many of these functions have been elucidated, but plenty of open questions remain. Recently, new functions of astrocytes have been disclosed—we briefly summarize advances in astrocyte-to-neuron conversion.

CNS damage and neuronal loss is the ultimate cause of several neurological disorders, including spinal cord injury, Alzheimer’s disease, Parkinson’s disease, and stroke [1]. Unlike peripheral tissues and organs, the mature mammalian neurons of the CNS are virtually non-regenerative. Sustained dysfunction of neurons leads to irreversible brain damage and severe functional consequences. Traditional cell therapy requires neuronal delivery to the patient’s brain by surgical means, however, the low neuronal survival rate limits its effectiveness. The direct conversion of somatic cells into neurons *in vivo* could be a promising approach of solving this problem.

Glial cells are auspicious candidates to facilitate reprogramming and differentiation into neurons thereby achieving *in situ* regeneration of neurons. Their conversion was first described by Götz *et al*. in 2002, reporting PAX6-induced reprogramming of astrocytes into neurons. In the meantime, many reports have been published on cell–cell transition, aimed at developing strategies for treatment of neuronal loss. Successful *in situ* conversion of glial cells into cerebral neurons has been demonstrated in some ground-breaking studies using a single neural transcription factor [2]. In a mouse model of ischemic stroke, specific overexpression of NeuroD1 in astrocytes induces the local conversion of astrocytes to neurons in areas of brain injury. Simultaneously, undifferentiated astrocytes undergo mitosis and proliferation, thus maintaining a relatively stable population. These converted neurons are integrated into the surviving nervous system with repetitive action potentials and robust synaptic activity [3]. More recently, it has been found that the induction of an increase in endogenous SOX2 in spinal cord injury caused conversion of NG2^+^ glia cells to DCX (a marker of neuronal regeneration)-positive cells, further development into mature neurons was not observed. Overexpression of exogenous SOX2 successfully transformed NG2^+^ glial cells into functional neurons and promoted functional recovery in mice [4]. Differential proteomic analysis of the neuronal and astrocytic mitochondrial composition enabled improvement of the conversional efficiency through clustered regularly interspaced short palindromic repeat activation (CRISPRa)-mediated transcriptional engineering [5]. Early dCas9-mediated upregulation of genes encoding (particularly neuron-enriched) mitochondrial proteins drastically improved the efficiency of glia-to-neuron conversion and neuronal survival, suggesting a driving role of mitochondrial proteins in this conversion.

**Figure 1.**
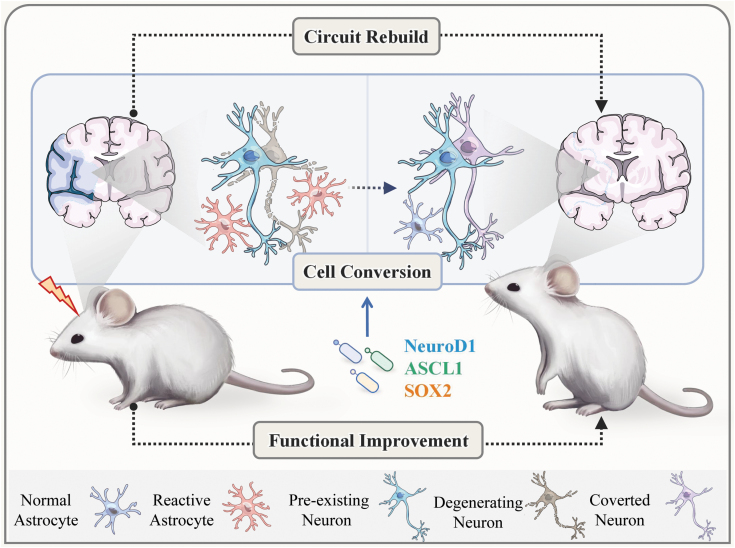
**Glia-to-neuron conversion restores lost neuronal functions after injury.** During CNS damage or neurodegenerative diseases, glial cells, such as microglial cells or astrocytes, are activated due to subsequent inflammatory responses. Overexpression of exogenous transcription factors (NeuroD1, ASCL1, SOX2) in glial cells allows their conversion into functional neurons, which are able to integrate into the neural network by establishing synaptic connections with endogenous neurons. The neurogenic reprogramming of glial cells enhances neural regeneration and promotes functional improvement in rodents and primates.

The glial cell conversion approach has attracted considerable attention in the field of neuroscience, in particular with regard to the potential of the transcription factor NeuroD1. Gong Chen and coworkers investigated the suitability of this factor for *in situ* neuronal regeneration in different neurological disease models: An astrocyte-to-neuron conversion rate of 80% was reported in the cerebral cortex of Huntington model mice, and more than 50% of the transformed neurons were DARPP32^+^ medium spiny neurons. Behavioral analysis revealed significant lifespan extension and improved motor function of NeuroD1 and Dlx2-treated R6/2 mice [6]. In monkeys, NeuroD1-mediated *in situ* conversion of astrocytes to neurons successfully renewed a large number of functional neurons after ischemic injury. The utilization of NeuroD1 adenovirus (AAV)-based gene therapy regenerated one-third of the total neuronal loss caused by ischemic injury and protected the impaired neurons, thus achieving important neuronal recovery [3, 7]. NeuroD1-carrying AAV also initiated *in situ* conversion of astrocytes into functional neurons in short- or long-term injured mouse spinal cord. In parallel, it was demonstrated that different transcription factor combinations could be used to control the converted neuronal subtypes and their ratios for precise neural regeneration and repair [8] (Fig. 1).

However, a recent *in vivo* study questioned the feasibility of the glia-to-neuron conversion in mice. Overexpression of NeuroD1 or knockdown of Ptbp1 did not facilitate astrocyte-to-neuron conversion using a lineage tracing method. Converted neurons were identified as pre-existing neurons in the brain, and the study failed to confirm astrocyte-to-neuron conversion [9]. Responding to these concerns, Chen Gong and coworkers demonstrated that a high viral titer of over 10^13^ GC/mL is prone to severe neuronal leakage, which, to some extent, renders the complex process of the *in situ* neuronal conversion unreliable. It is claimed that astrocytes under lineage trace are more difficult to convert than normal glial cells, and that increased NeuroD1 expression with enhancers is required to overcome the resistance to conversion and to achieve *in situ* neural regeneration [10].

Undoubtedly, introduction and validation of these conversion techniques provide an important strategy for the treatment of neural injury and degenerative lesions, which (hopefully) will enable reconfiguration of neural circuits and restoration of brain function (Fig. 1).

A possible limiting factor in astrocyte-to-neuron conversion is the availability of astrocytes. Patients suffering from severe injury that causes substantial tissue loss accompanied by drastic astrocyte and neuron decline may require additional treatment to preserve local astrocytes for neuronal conversion. Cell transplantation could be needed to compensate the tissue defect in the CNS prior to astrocyte-to-neuron transformation.

There is another challenge: different brain regions can be involved in the neuronal loss during injury or neurodegenerative diseases, such as the cortex, striatum and hippocampus in the MCAO model. Consequently, different transcription factors may be required to achieve conversion of astrocytes to neurons of different subtypes for effective repair in distinct brain regions. Even after successful neuronal conversion, it is questionable whether the newly generated neurons in different brain regions can form correct connections and serve as substitute for lost neurons. Experiments in adult mice suggest that the converted neurons are able to integrate into pre-existing brain circuits, but clinical application will require painstaking translational research—which is likely to be a long way to go.
